# Prostate Cancer and Aspirin Use: Synopsis of the Proposed Molecular Mechanisms

**DOI:** 10.3389/fphar.2017.00145

**Published:** 2017-03-21

**Authors:** Nadeem Bilani, Hisham Bahmad, Wassim Abou-Kheir

**Affiliations:** Department of Anatomy, Cell Biology and Physiological Sciences, Faculty of Medicine, American University of BeirutBeirut, Lebanon

**Keywords:** prostate cancer, NSAIDs, aspirin, COX pathway, chemoprevention

## Abstract

**Background:** Prostate cancer (PCa) is a critical health burden, impacting the morbidity and mortality of millions of men around the world. Most of the patients with PCa have their disease at first sensitive to androgen deprivation treatments, but later they develop resistance to therapy and eventually die of metastatic castration-resistant prostate cancer (CRPC). Although the newly developed anti-androgen therapies are effectively alleviating symptoms and prolonging lives of patients, there are still no curable treatments for CRPC. Recently, statistical studies have shown that the chronic use of aspirin might be significantly associated with better outcomes in PCa patients. Through this review, we aim to identify the different proposed molecular mechanisms relating aspirin to the pathobiology of PCa neoplasms, with a major focus on basic research done in this context.

**Methods:** Articles were retrieved via online database searching of PubMed and MEDLINE between 1946 and September 2016. Keywords and combinations related to PCa and aspirin were used to perform the search. Abstracts of the articles were studied by two independent reviewers and then data extraction was performed on the relevant articles that met our review objectives.

**Results:** Aspirin, a non-steroidal anti-inflammatory drug (NSAID), affects the proliferation, apoptosis, resistance and metastasis of PCa cell lines, through both COX-dependent and COX-independent mechanisms. It also lowers levels of the PCa diagnostic marker prostate specific antigen (PSA), suggesting that clinicians need to at least be aware if their patients are using Aspirin chronically.

**Conclusion:** This review strongly warrants further consideration of the signaling cascades activated by aspirin, which may lead to new knowledge that might be applied to improve diagnosis, prognosis and treatment of PCa.

## Introduction

Prostate cancer (PCa) is the most prevalent solid tumor in men from industrialized nations and is the second largest cancer-related killer (Center et al., 2012; Siegel et al., 2013). Age is a significant risk factor for the disease. The incidence in USA jumps from 1 in 7,964 to 1 in 8 when comparing men under 40 years of age with men older than 70, respectively (Siegel et al., 2013). Most of the patients with advanced PCa are initially sensitive to traditional treatments of androgen ablation therapy. This is the mainstay of treatment, and leads to the regression of PCa tumors (Suzuki et al., 2000; Feldman and Feldman, 2001). However, with the progression of the disease, PCa often develops resistance to therapy and patients may eventually die of this metastatic castration-resistant prostate cancer (mCRPC). As many as 50% of PCas will progress from an androgen-dependent (AD) to a hormone refractory state of disease, and will metastasize to bone and pelvic lymph nodes (Thalmann et al., 1994).

There are still no curable treatments for CRPC (Karantanos et al., 2013). Drugs such as abiraterone, enzalutamide and TOK-001, bone-targeted therapies (such as bisphosphonates, denosumab, and Radium-223), and immunotherapies all have questionable efficacy (Chaturvedi and Garcia, 2014). Hence, novel treatment strategies are a necessity to improve the quality and span of life for PCa patients. Understanding the underlying mechanisms behind progression of PCa to CRPC and its metastasis is crucial in defining new therapeutic targets and prophylactic therapies for this type of cancer.

While the role of non-steroidal anti-inflammatory drugs (NSAIDs) in preventing colorectal cancer has been well-established (Muscat et al., 1994), numerous epidemiological studies have shown that they are protective against other cancers as well (Baron and Sandler, 2000; Cha and DuBois, 2007). One study reported nearly a 63% drop in the relative risk for colon cancer, 39% for breast cancer, 36% for lung cancer, and 39% for PCa with the increasing intake of NSAIDs (mainly aspirin or ibuprofen; Harris et al., 2005). One meta-analysis looked specifically at the association between aspirin and PCa, combining the results of 39 studies (20 case-control and 19 cohort studies; Liu et al., 2014). It was found that aspirin use was significantly associated with lower PCa incidence (OR = 0.92, 95% CI = 0.87–0.97) and lower PCa-specific mortality (HR = 0.86, 95% CI = 0.78–0.96). While evidence suggests a protective effect of aspirin, the processes underlying this remain unclear.

The aim of this paper is to review some of the proposed mechanisms relating aspirin to the pathobiology of PCa neoplasms, with a main focus on the basic science research done in this context.

## Decreased PSA levels in chronic aspirin users among prostate cancer patients

The majority of PCa patients first learn they might have the disease through a blood test that looks for increased or rising levels of PSA protein (Hamilton et al., 2008), produced by luminal cells in the prostate (Feldman and Feldman, 2001). In these settings, PSA can indicate the presence or recurrence of PCa. The concern is that drugs that artificially lower PSA levels might mask this marker, which normally flags the development of a prostate neoplasm. One study analyzed PSA levels in a cohort of over 1,000 men and found that PSA levels in the sample of men taking aspirin were nearly 10% lower than a control sample not taking aspirin (Hamilton et al., 2008). Researchers question whether this decrease in PSA is artificial or whether it might be a direct result of anti-tumorigenic properties of aspirin. Evidence points in both directions.

Multiple epidemiological studies have reported an increase in high-grade PCa (HGPCa) in aspirin users (Bosetti et al., 2012; Olivan et al., 2015). One suggested explanation found in the literature is that aspirin and other NSAIDs lower PSA levels, but is not itself anti-tumorigenic (Berg et al., 2009; Schroder et al., 2009). If true, this would likely delay diagnosis of the disease, allowing it to progress unnoticed. Alternatively, it was also suggested that these drugs might relieve cancer-associated pain that normally motivates patients to investigate their symptoms earlier on. In contrast, it must also be noted that other studies show decreased levels of HGPCa in chronic aspirin users (Brasky et al., 2010; Dhillon et al., 2011). More conclusive research needs to be conducted to determine the mechanism by which aspirin decreases PSA: whether this is a by-product of the drug's anti-tumorigenic properties or truly a masking effect. Nonetheless, physicians should be weary of the accuracy of the PSA blood test in patients taking aspirin chronically.

## Aspirin and the COX pathway in prostate cancer

**Figure 1:**
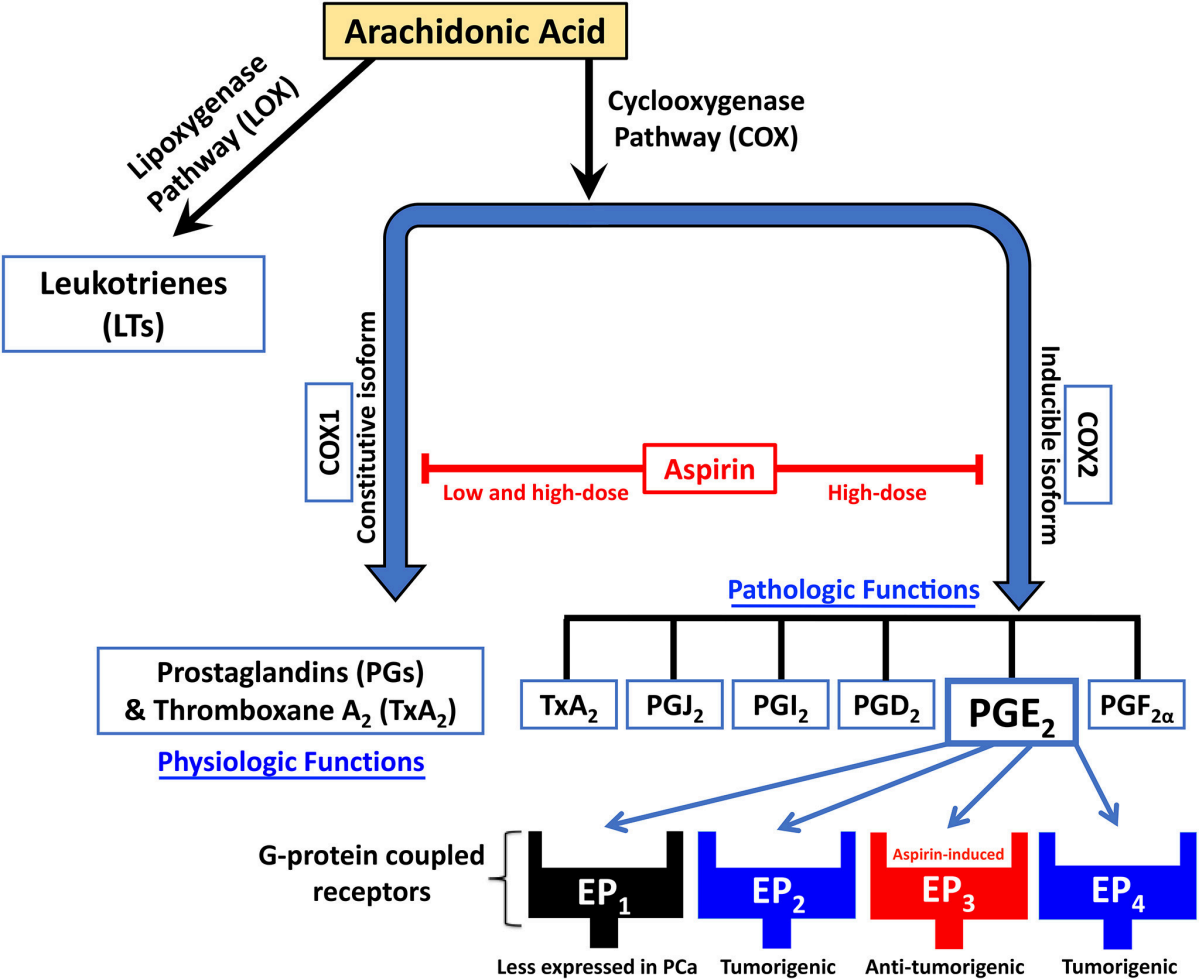
**Schematic diagram showing the mechanism of action of NSAIDs like aspirin in inhibiting the metabolism of arachidonic acid by blockade of the cyclooxygenases (COX) pathway and the prostaglandins (PG) synthase pathway, thus suppressing PGs synthesis**. Aspirin also works by upregulating EP_3_, an inhibitory G-protein-coupled receptor of the prostaglandin PGE_2_.

NSAIDs inhibit the metabolism of arachidonic acid by blocking the cyclooxygenases (COXs) pathway and the prostaglandins (PGs) synthase pathway, thus suppressing PG synthesis and inflammation (Majima et al., 2003; Figure 1). COXs are key enzymes in prostanoid synthesis, existing in two isoforms: COX1 and COX2. COX1, referred to as “constitutive isoform,” is expressed in several tissues under basal conditions. COX2 is believed to be undetectable in normal human tissues, but can be induced by mitogens, cytokines, and tumor promoters under various, mainly pathological condition. It is thus referred to as “inducible isoform” (Katori and Majima, 2000; Gupta and Dubois, 2001; Subbaramaiah and Dannenberg, 2003). COX2 activation hence promotes enhanced PGs synthesis in both inflamed and neoplastic tissues (Bennett, 1986; Rigas et al., 1993).

Aspirin is unique in that it irreversibly blocks both COX1 and COX2 activities through acetylation of significant enzyme serine residues. Ergo, new COX activity can only be achieved following aspirin treatment through *de novo* synthesis of COX. The main mechanism by which NSAIDs are thought to prevent the growth of neoplasms is the blocking of COX2 activity (Thun et al., 2002), though studies have shown that NSAIDs like aspirin have anticancer effects through both COX-dependent and independent cascades (Grosch et al., 2006; Alfonso et al., 2014).

Several studies have demonstrated higher expression of COX2 in PCa tumor tissues than in benign prostate tissues (Gupta et al., 2000). It has been shown that both LNCaP and PC3 PCa cell lines express COX2. High COX2 expression in PCa cells has also been associated with poor prognosis (Khor et al., 2007). This has further corroborated the suggestion that NSAIDs could play a role in reducing PCa risk specifically through inhibiting the COX pathway.

*In vivo*, anti-inflammatory doses of aspirin (a daily dose of >2,000 mg) do bring systemic concentrations high enough to inhibit both COX1 and COX2. However, in nucleated cells, due to *de novo* synthesis, inhibition can only be prolonged with repeated daily dosing (Thun et al., 2012). It has been suggested in that same paper that aspirin in lower doses might still effectively inhibit COX2 due to partial dependence of COX2 expression in monocytes on activated platelets. Consequently, aspirin permanently inactivates COX in platelets, thus indirectly inhibits COX2 expression (Thun et al., 2012).

The blockage of COX prevents the production of downstream PG products, known as prostanoids, such as TXA_2_, PGI_2_, PGE_2_, PGF_2α_, and PGD_2_. These prostanoids have roles in decreasing apoptosis and increasing cellular proliferation (Thun et al., 2012). One PCa-specific study reported that aspirin-treated LNCaP and PC3 PCa cells had the same proportion of dead cells as non-treated cells, signifying that aspirin might not induce apoptosis but instead suppresses proliferation (Olivan et al., 2015). The literature is not conclusive on this, however. In addition, this paper reported decreased colony formation and significant inhibition of invasion and migration capacities in aspirin-treated cells (PC3 cells in particular) with higher effects when aspirin is combined with simvastatin, a cholesterol-lowering drug (Olivan et al., 2015).

Among the five PGs that have been identified in the COX pathway, PGE_2_ is the most common and ubiquitously produced PG, contributing to tumorigenesis via cell proliferation induction (Tjandrawinata et al., 1997), angiogenesis (Wang and Klein, 2007; Jain et al., 2008), invasion (Sheng et al., 2001; Buchanan et al., 2003), and metastasis (Konturek et al., 2005; Fulton et al., 2006). PGE_2_ levels are 10-fold higher in human malignant PCa tissues than in benign prostatic tissues (Chaudry et al., 1994). PGE_2_ works through EP_1_, EP_2_, EP_3_, and EP_4_, four G-protein coupled receptors (Kashiwagi et al., 2013). Human prostate epithelial cells express EP_2_ and EP_4_ receptors, while EP_1_ and EP_3_ receptor expression in these cells is not detected (Wang and Klein, 2007). EP_3_ is distinct from EP_2_ and EP_4_ in that it is not a stimulatory but instead an inhibitory G-protein. Thus, EP_3_ decreases levels of the secondary messenger cAMP when activated. A study by Kashiwagi et al. reported that aspirin decreases Androgen Receptor (AR) mRNA and protein levels in dose-and time-dependent manners (Kashiwagi et al., 2013), which is thought to be related to the proliferation of PCa. Interestingly, the same study reported upregulation of EP_3_ expression and a consequent downregulation of AR and EP_2_ expression in PCa cell lines upon aspirin treatment. This domino effect was confirmed using both pharmacological and knockdown methods. The results are supported by another study that found that EP_3_ signaling inhibits the NF-κB pathway (Wang et al., 2010), which decreases AR expression levels in PCa cells (Zhang et al., 2009).

This was not the first paper to claim this connection to the NF-kB pathway. Lloyd et al. previously showed that aspirin inhibits NF-κB, resulting in diminished urokinase-type plasminogen activator (uPA) secretion—one of the crucial molecules involved in cancer metastasis—from the highly invasive human PC3 PCa cells (Lloyd et al., 2003). The inhibition of COX in platelets might also be significant, since experimental evidence has shown that platelets are significant in cancer metastasis through the blood (Labelle et al., 2011; Dudeja et al., 2012). This effect is mediated through their ability to aggregate and allow cancer cells to escape immune detection as well as the pro-angiogenic factors, such as VEGF, that they release (Usman et al., 2015). Thus, the EP_3_ receptor might represent a potential molecular target for developing therapy in PCa.

## Aspirin and COX-independent regulation of the cell cycle in prostate cancer

Aspirin might influence regulation of the cell cycle, which is dependent on a family of proteins called cyclins and another group of protein kinases called cyclin-dependent kinases (CDKs). When combined with statins in treatment, aspirin was shown to decrease proliferation of LNCaP cells with a reduction in cyclin D1 levels—which modulates cell cycle progression (Olivan et al., 2015). Aspirin, on its own, was shown to cause ubiquitin-dependent degradation of cyclin D1 in colorectral cancer cells (Thoms et al., 2007). Further research is needed to deduce whether aspirin can instigate the same mechanism in PCa cells in the absence of statins, since on their own, statins were also shown to be associated with low expression of cyclin D1 in a breast cancer trial (Feldt et al., 2015).

Another proposed mechanism of control—as suggested by epidemiological studies in colorectal cancer patients (Seiler, 2003; Laukaitis and Gerner, 2011)—is the induction of polyamine catabolism and subsequent regulation of cell proliferation and cancer progression (Arisan et al., 2014). Polyamines are small cationic molecules, formed from the decarboxylation products of ornithine and S-adenosyl-methionine. They are present in high concentrations in rapidly dividing tumor cells (Agostinelli et al., 2010). Although intracellular levels of polyamines are elevated in normal prostate gland (Karr et al., 1991), abnormal regulation of their metabolism results in rapid cell proliferation and PCa progression (Arisan et al., 2014). In fact, when PCa cells were treated with CDK inhibitors purvalanol and roscovitine, which induce apoptosis by promoting cell cycle arrest in cancer cells, upregulation of polyamine catabolic enzymes (SSAT, SMO, and PAO) was induced. This caused the depletion of intracellular polyamine levels (Arisan et al., 2014). In the same study, silencing of SSAT prevented CDK inhibitors-induced apoptotic cell death in PCa cells (Arisan et al., 2014). Accordingly, aspirin has been recognized as an inducer of SSAT by allowing NF-κB binding on the *Sat1* gene (Babbar et al., 2006). However, another study showed that treating LNCaP PCa cells with aspirin decreased induced SSAT activity in these cells (Li et al., 2016). Authors of this study concluded that SSAT and its related polyamine metabolism may play a significant role in the susceptibility of PCa to aspirin therapy (Li et al., 2016). The potential relevance of these mechanisms needs to be further explored, especially using *in vivo* trials and feasible, non-toxic doses of aspirin.

One study found that aspirin promotes “tumor necrosis factor-related apoptosis inducing ligand” (TRAIL)-induced apoptosis in both androgen-dependent LNCaP cells and other LNCaP derived cells (C4, C4-2, and C4-2B), which represent CRPC, through decreased survivin protein—a versatile modulator of cell division and apoptosis in cancer (Altieri, 2003)—expression in these cells (Yoo and Lee, 2007).

And finally, researchers have also evaluated the effect of new nitric oxide (NO) donating NSAIDs, including NO-aspirin and NO-ibuprofen, on LNCaP and PC3 PCa cell lines. They found these drugs to be potent inhibitors of proliferation and inducers of apoptosis via enhanced caspase-3 expression (Royle et al., 2004). One reason for the importance of those novel NSAIDs over classical ones lies in the presence of NO, which when endogenous, contributes to the action of immune cells against foreign pathogens and tumor cells. NO was additionally suggested to play a role in the modulation of cell death by apoptosis, though this effect depends on a multitude of factors, including the concentration of NO and the cell type (Wallace and Soldato, 2003). Interestingly, NO-aspirin had been shown to be much more potent, even at lower concentrations, at inducing apoptosis and inhibiting proliferation in those PCa cells than conventional aspirin (Royle et al., 2004). In accordance, NO-aspirin inhibited proliferation of PC3 and DU145 PCa cells through blocking Wnt/β-catenin signaling in those cells (Lu et al., 2009).

Other studies have implicated the lipoxygenase (LO) pathway of arachidonic acid metabolism in the progression of PCa. Yang et al. showed that LO products, including 12-HETE, were significantly higher in malignant prostate tissue than non-malignant tissue. The role of an NSAID like aspirin in this process is unclear (Yang et al., 2012). While one might intuitively warn that the inhibition of COX redirects arachidonic acid to the LO pathway, the evidence in the literature is not conclusive. Gray et al. looked at COX and LO activity in whole blood, noting that the blocking of COX was not actually associated with an increase in LO products. Furthermore, they reported that NO-aspirin even reduced LO activity, a notion supported by other publications as well (Gray et al., 2002). Brunn et al. for instance, reported that endogenously released NO inhibits the production of 5-LO metabolites in macrophages (Brunn et al., 1997). However, these precise mechanisms of NO-aspirin still remain the subject of investigation.

Thus, with the failure of androgen ablation therapies and emergence of hormone-refractory states in PCa, enhancing tumor cell death via facilitating apoptosis of cancer cells using aspirin may be an effective promising chemopreventive therapy for the disease in the future.

## Aspirin and COX-independent regulation of metastasis of prostate cancer cells

Aspirin treatment has been associated with decreased migration of PCa cell lines and increased levels of α2 integrin (Olivan et al., 2015), which may be a metastasis suppressor as suggested by Ramirez et al. (2011). These results are controversial, however, as other studies reported conflicting data. Other literature found that the expression of integrin α2β1 actually induces PCa metastasis to the bone (Hall et al., 2008; Van Slambrouck et al., 2009; Sottnik et al., 2013). These studies suggest that the expression of this protein is in fact correlated with the different stages of cancer progression (Hall et al., 2008; Van Slambrouck et al., 2009; Mitchell et al., 2010). This might be the cause of reported higher levels of HGPCa in patients treated chronically with aspirin (Olivan et al., 2015); however, further research is needed to clarify the role of integrins in PCa tumors and whether they can be a molecular target for therapy.

It has been demonstrated that cell migration, and the process of cancer metastasis, is regulated or influenced by different molecular mechanisms. Another mechanism explored specifically in regards to PCa concerns p75^NTR^, a member of the tumor necrosis factor (TNF) receptor superfamily and tumor suppressor highly expressed in normal prostate epithelial cells (Chao, 1994). This high expression diminishes as the tumor progresses (Pflug et al., 1992). Reports have shown that NSAIDs like aspirin induce p75^NTR^ expression through the p38 mitogen-activated protein kinase (MAPK) pathway (Wynne and Djakiew, 2010). Correlating with the induction of p75^NTR^ by NSAIDs is the induction of Nag-1, a member of the TGF-B superfamily that inhibits cell migration, possibly through blocking the activity of uPA, and matrix metalloproteinases MMP2 and MMP9 (Wynne and Djakiew, 2010). Thus, Wynne and Djakiew et al. proposed that NSAID suppression of cell migration might be mediated by Nag-1 induction, downstream of p75^NTR^.

## The effect of aspirin on chemotherapy in prostate cancer

Aspirin may promote resistance to treatment in PCa in three different mechanisms. First, many chemotherapeutic agents work by targeting rapidly-dividing cells, thus decreased cellular proliferation—as was shown to be an effect of aspirin on PCa cells—might decrease the efficacy of these anticancer treatments.

Second, one study outlined how aspirin might produce resistance against chemotherapy by looking at how the drug affects P-glycoprotein (P-gp) expression (Rotem et al., 2000). It was found that aspirin and similar drugs induce protein kinase C (Zhu et al., 1999; Zimmermann et al., 2000), which enhances the activity of a nuclear factor for IL-6 expression (Trautwein et al., 1993; Combates et al., 1997). This consequently increases the activity of the MDR1 promoter (Combates et al., 1994). MDR1 encodes for an efflux pump called P-gp, which removes a number of anticancer drugs from the cell, thereby causing the chemotherapy agent to be ineffective at normal concentrations. Rotem et al. concluded that although aspirin reduces cellular proliferation in all 3 PCa cell lines studied (DU-145, PC-3, and LNCaP), it induces a three-fold increase in the percentage of cells expressing P-gp in LNCaP cell lines on the other hand (Rotem et al., 2000). This expression of P-gp was reversible, only persisting around 3 days, implying that it was not mediated by changes at the genetic level (Rotem et al., 2000).

Third, it has been noted that aspirin causes cells to become more thermotolerant by increasing heat shock protein (HSP)-70 expression in these cells (Amici et al., 1995). The administration of this drug might thus also interfere with hyperthermic treatment, which is commonly used in conjunction with chemotherapy or radiotherapy to enhance the effectiveness of these forms of treatment.

## Conclusion and perspectives

While large epidemiological studies have significantly shown an inverse correlation between aspirin intake and cancers like PCa, tests and assays using cell lines have revealed desirable and undesirable outcomes that need to be explored more thoroughly. It is thus clear that there are many reasons why clinicians need to at least be aware if their patients with PCa are taking aspirin. Whether or not aspirin can be used as an adjuvant to therapy for PCa is yet undecided. This review strongly warrants further consideration of the signaling cascades activated by the aspirin (Table 1), which may lead to new knowledge that might be applied to improve the diagnosis, prognosis and treatment of PCa (Figure 1).

**Table 1:** **Proposed molecular mechanisms relating aspirin use in prostate cancer**.

	**Mechanism of Action**	**References**
Cancer detection	Decreases PSA mRNA and protein levels	Kashiwagi et al., 2013
Regulation of proliferation/apoptosis	Inhibition of SSAT	Li et al., 2016
	Reduction in cyclin D1	Olivan et al., 2015
	Decreases AR, upregulation of EP_3_ and downregulation of EP_2_	Kashiwagi et al., 2013
	Blocks Wnt/B-catenin signaling	Lu et al., 2009
	Decreases survivin expression, TRAIL-induced apoptosis	Yoo and Lee, 2007
	Enhances caspase 3 expression	Royle et al., 2004
	Inhibition of NF-κB pathway and decrease in AR expression	Lloyd et al., 2003
Regulation of metastasis	Increases a2-integrin expression	Olivan et al., 2015
	Inhibition of NK-kB pathway and decrease in uPA expression	Lloyd et al., 2003
	Induction of p75^NTR^ through MAPK pathway and Nag-1	Wynne and Djakiew, 2010
Resistance to treatment	Increases expression of MDR1	Rotem et al., 2000
	Increases HSP-70 expression	Amici et al., 1995

*SSAT, spermidine/spermine N(1)-acetyltransferase; AR, androgen receptor; EP_3_, E-type prostaglandin receptor 3; EP_2_, E-type prostaglandin receptor 2; TRAIL, tumor necrosis factor-related apoptosis inducing ligand; NF-κB, nuclear factor kappa-light-chain-enhancer of activated B cells; uPA, urokinase-type plasminogen activator; p75^NTR^, p75 neurotrophin receptor; MAPK, mitogen-activated protein kinase; Nag-1, N-acetylglucosamine-1; MDR1, multidrug resistance protein 1; HSP-70, heat shock protein 70*.

## Author contributions

All authors listed were involved in the concept, literature screening, and writing of the article, and approved it for publication.

## Funding

This research was supported by funding from the Medical Practice Plan (MPP) at AUB-FM. The funders had no role in study design, data collection and analysis, decision to publish, or preparation of the manuscript.

### Conflict of interest statement

The authors declare that the research was conducted in the absence of any commercial or financial relationships that could be construed as a potential conflict of interest. The reviewer JL and handling Editor declared their shared affiliation, and the handling Editor states that the process nevertheless met the standards of a fair and objective review.
